# Online bipartite matching methodology for anti-epidemic resources allocation: an adaptive time window based on reinforcement learning

**DOI:** 10.3389/fpubh.2025.1644499

**Published:** 2026-01-08

**Authors:** Zhiyong Wu, Sulin Pang, Suyan He

**Affiliations:** 1 School of Digital Economics and Trade, Guangzhou Huashang College, Guangzhou, China; 2 Institute of Finance Engineering/School of Emergency Management, Jinan University, Guangzhou, China; 3 Department of Economics, Guangdong Institute of Public Administration, Guangzhou, China

**Keywords:** major epidemics, bipartite matching, adaptive time window, resource allocation, reinforcement learning

## Abstract

**Background:**

This study aimed to investigate the online matching problem for anti-epidemic resources among multiple suppliers and recipients in the Internet of Healthcare System during a major outbreak. It accounts for the heterogeneity of supply and demand.

**Methods:**

A multi-stage online dynamic bipartite matching model based on time windows is developed, which can be reformulated as a Markov decision process. An adaptive time window batch bipartite matching algorithm based on reinforcement learning is proposed, which utilizes the nearest neighbor's first heuristic strategy to allocate anti-epidemic resources.

**Results:**

The optimal window size in *fixed time window batch matching strategy* (FTWBM) outperforms the results of *adaptive time window batch matching strategy* (ATWBM). However, the ATWBM strategy demonstrates greater effectiveness in adapting to the dynamic changes in epidemic prevention and control, particularly in partially optimistic scenarios.

**Conclusions:**

The results revealed that, although the average matching rate consistently increases, the average waiting time initially decreases before rising again as the matching time window expands. This finding implies that health operations managers should modify the matching time window in response to changing epidemic dynamics and resource availability.

## Introduction

Major epidemics have consistently posed significant threats to human health and presented challenges to public health systems. They serve as a substantial test of governance structures and capacities (1). China is actively promoting the integration of information technologies with medical and healthcare services. This initiative has sparked a new wave of technological and management innovations, interconnected through the Internet of Healthcare Systems (HIS) and characterized by intelligent medical and healthcare management. Internet of Things (IoT) technology is utilized to monitor the utilization of medical resources, and corresponding resource allocation plans to meet demand are proposed (2). These sharing platforms have played a crucial role in helping individuals understand the dynamics of the epidemic and alleviate anxiety, as evidenced by their significant contributions to enhancing social emergency management capabilities during the pandemic.

Simultaneously, major epidemics create a severe and complex situation characterized by rapid and widespread transmission, which can lead to insufficient preventive measures and resource constraints. This has heightened the challenges associated with epidemic prevention and swift response. Consequently, the rapid advancements in smart medical technology and healthcare, the implementation of IoT technology, and the emergence of sharing platforms to facilitate the effective allocation of preventive resources have become critical demands in public health.

This study examines the online matching problem for anti-epidemic resources among multiple suppliers and recipients from the onset to the conclusion of major epidemic emergencies. The supply of anti-epidemic resources remains relatively stable over a short time frame and does not exceed the existing delivery capacity. However, it is constrained by the geographical scope of service. Typically, during an epidemic, the demand for anti-epidemic resources experiences a sudden surge, whereas the quantity supplied often falls short of the quantity demanded. Furthermore, suppliers and recipients exhibit heterogeneity. This study addresses three key questions: (1) Which strategy should be employed for matching anti-epidemic resources: instant matching or delayed batch matching? (2) If the delayed batch matching strategy is adopted, should the fixed time window matching strategy or the adaptive time window matching strategy be selected? (3) If the delayed batch matching strategy is implemented, what is the impact of the size of the matching time window on the outcomes of the matching decision?

In this study, we applied the matching model to address the allocation of anti-epidemic resources and propose a multi-stage online dynamic bipartite matching model (MSODBM). We transformed the problem into a Markov decision process and designed a reinforcement learning-based adaptive time window batch bipartite matching algorithm that employs the nearest neighbor first (NNF) heuristic strategy. The principal contributions can be summarized as follows: First, a multi-stage dynamic bipartite matching model for anti-epidemic resources is proposed to address online allocation problems with resource constraints. This model effectively integrates the sharing organization framework with the challenge of allocating anti-epidemic resources, representing a novel application of intelligent technology in this field. The traditional allocation of anti-epidemic resources mostly focuses on optimizing allocation at the group level through offline methods. Due to information asymmetry, it is difficult to achieve an efficient allocation of anti-epidemic resources at the individual level. The online approach based on sharing platforms provides new possibilities for resource allocation at the individual level. Second, an adaptive time window batch bipartite matching approach is proposed. Drawing on the exploration-exploitation trade-off concept from reinforcement learning, we use the minimum average waiting time as the learning reward and employ a heuristic strategy that prioritizes the nearest neighbor. Given unpredictable epidemic developments and dynamic fluctuations in supply and demand, there is no universally optimal or static matching time window. Maintaining a relatively high level of exploration for the matching time window plays a significant role in the effective allocation of resources. Third, a Hungarian-based many-to-many matching algorithm with capacity constraints is developed. The classic Hungarian algorithm primarily addresses the assignment of a single task or resource without accounting for the matching capacity or the many-to-many matching problem. Given the mismatch between the supply and demand of anti-epidemic resources, which constitutes a many-to-many matching challenge, it is essential to extend the classic Hungarian algorithm. This extension is achieved by incorporating a capacity attribute and refining the augmented-path search mechanism.

The remainder of this study is organized as follows. In Section 2, we review related work. Section 3 formally defines the problem. In Section 4, a multi-to-multi bipartite matching heuristic strategy and an algorithm based on a self-adaptive time window are proposed. Extensive experiments conducted on synthetic data are presented in Section 5. Finally, Section 6 provides managerial insights and outlines potential directions for future research.

## Related literature

This study primarily engages with three streams of literature. The first stream is bipartite matching theory, also known as bipartite graph matching, which was originally developed to address the issue of inseparability in resource allocation (3, 4). This theory has been applied to design matching mechanisms across various markets (5, 6), including medical and healthcare, labor markets, school selection mechanisms, and kidney exchange markets. Roth (7) was the first to introduce the concept of bipartite matching and to define the terms “bipartite” and “bipartite matching.” A series of research findings has emerged based on the one-sided vertex arrival model (8). Some scholars have explored the bipartite matching problem under sequential arrival conditions (9), edge-weighted online bipartite matching problems (10), non-stationary online bipartite matching problems (11), and online matching problems with random rewards (12). In the current literature, user arrival information is categorized into adversarial and Bayesian arrivals based on the sequence of users arriving online. The occurrence of major epidemics is often sudden, leading to uncertainty in the timing and quantity of information from suppliers and recipients. Consequently, the online matching problem is characterized as an adversarial complete online matching problem.

The second stream is the sharing economy. The sharing economy enhances the efficiency of utilizing decentralized private assets by facilitating large-scale collaborative activities among individuals (13). Serving as a link among suppliers and recipients, sharing platforms integrate offline idle items, labor, and resources, enabling effective matching and trading of resources, which ultimately leads to profit generation. These platforms employ location-based technologies and algorithms to search for and match resources, providing users with information sorted by these algorithms in real time (14). An efficient recommendation system, supported by big data analysis and machine learning, improves matching efficiency and helps alleviate the “information problem” (13). Existing studies provide valuable insights for addressing online matching challenges.

The third stream focuses on emergency resource allocation during public health events. In the event of a public health emergency, the most affected areas may encounter various challenges due to insufficient medical personnel and supplies (15). Given the constraints of limited resources, a critical issue is how to allocate these resources effectively across different areas of demand to control the epidemic as swiftly and effectively as possible (16). To enhance the effective allocation of emergency resources, some researchers have developed collaborative emergency medical resource allocation models driven by epidemic forecasts to address collaborative decision-making challenges with multiple objectives (17). A demand forecasting model with a general demand forecasting function was constructed to address the short-sighted behavior often observed in medical resource allocation (18). In response to the multi-regional Ebola outbreak, a two-stage model was established, taking into account a limited medical treatment team to determine both the timing of resource allocation decisions and the quantity of resources allocated (19). Considering the high volatility and time-varying characteristics of COVID-19, a multi-stage, multi-type online medical service model was developed (20). Additionally, some scholars have proposed a meta-population online resource allocation and epidemic transmission coupling model (21), examined the fairness of vaccine resource allocation (22), and analyzed the space-time competition of medical supply and demand (23).

Based on a review of the existing literature, this study identifies two significant research gaps. First, while some studies have examined decision-making in the allocation of emergency resources for public health crises, the focus has primarily been on predictive and optimized resource allocation methods. There is a scarcity of literature addressing the application of shared organizational models in the context of allocating resources for public health emergencies. Second, although new resource organization models based on sharing platforms have been widely adopted in sectors such as transportation, goods delivery, housing leasing, and online medical consultations, there is limited literature on the application of spatial-temporal online matching methods to allocate public health emergency resources. Furthermore, the emergence of resource allocation models based on sharing platforms presents new opportunities for the efficient allocation of anti-epidemic resources in the digital age.

## Model formulation

### Problem definition

**Figure 1:**
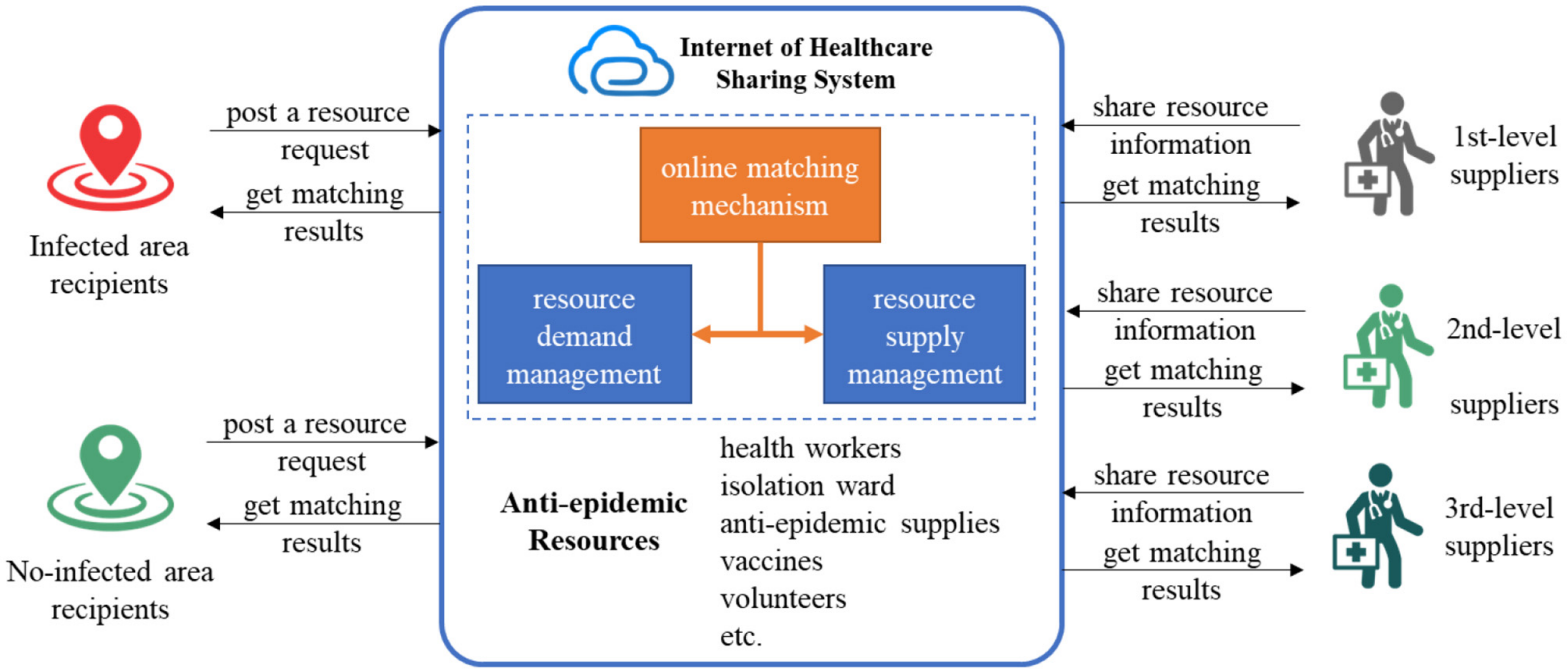
The HIS of anti-epidemic resource management.

The HIS platform connects individuals, objects, and their informational resources in both fixed and mobile environments through high-speed communication networks (24). This connectivity effectively coordinates the allocation of anti-epidemic resources. The online matching problem primarily involves pairing suitable resources from suppliers with recipients affected by the epidemic. The HIS allocates anti-epidemic resources among recipients and suppliers according to online matching rules, aiming to fulfill the needs for these resources as quickly and broadly as possible (Figure 1). In the pursuit of maximizing resource matching, the deployment time of anti-epidemic resources emerges as a critical influencing factor that plays a vital role in effectively managing the epidemic. Therefore, we concentrate on maximizing the matching of anti-epidemic resources while minimizing the total waiting time.

The recipient *i* ∈ ***D*** and the resource supplier *j* ∈ ***S*** dynamically arrive at HIS in a time series order. Recipient *i* can be represented by a quintuple *i* = (*id*_*i*_, *loc*_*i*_, *dr*_*i*_, *requesttime*_*i*_, *q*_*i*_), where *id*_*i*_ indicates a specific identifier, *loc*_*i*_ indicates the location, *dr*_*i*_ ∈ {*high, low*} indicates the epidemic prevention level, *requesttime*_*i*_ indicates the request time, and *q*_*i*_ indicates the required quantity. Supplier *j* is represented by *j* = (*id*_*j*_, *loc*_*j*_, s*r*_*j*_, *requesttime*_*j*_, *q*_*j*_, *l*_*j*_), where *id*_*j*_ indicates a specific identifier, *loc*_*j*_ indicates the location, *sr*_*j*_ ∈ {1, 2, 3} indicates the supply level, *requesttime*_*j*_ indicates the supply time, *q*_*j*_ indicates the quantity supplied, and *l*_*j*_ indicates the largest geographical range that *j* can be supplied.

Anti-epidemic resources are categorized into both reusable and non-reusable types. The resource matching set ***M*** encompasses all matching responses requested by both the supplier and the recipient. Each response can be represented by a quintuple (*i, j, q*_*ij*_, τ_*ij*_, *rate*_*ij*_), where *i* indicates the request from the recipient, *j* indicates the supplier capable of providing resources to *i*, *q*_*ij*_ indicates the quantity of resources that *j* can provide to *i*, τ_*ij*_ indicates the waiting time required for *i* to *j*, and *rate*_*ij*_ indicates the resource matching rate between *j* and *i*.

### Model formulation

The foundational model is articulated as follows.


$$\begin{array}{l} objective.max\;f\left(t\right)=\overset{D}{\underset{i}{\sum }}\overset{S}{\underset{j}{\sum }}q_{ij}\left(t\right)x_{ij}\left(t\right) \end{array}$$ (1)

$$\begin{array}{l} s.t.\;\overset{S}{\underset{j}{\sum }}q_{ij}\left(t\right)\leq q_{i}\left(t\right),\;i\in D \end{array}$$ (2)

$$\begin{array}{l} \overset{D}{\underset{i}{\sum }}q_{ij}\left(t\right)\leq q_{j}\left(t\right),\;j\in S \end{array}$$ (3)

$$\begin{array}{l} l_{ij}\leq l_{j} \end{array}$$ (4)

$$\begin{array}{l} \overset{S}{\underset{j}{\sum }}q_{ij}\left(t\right)x_{ij}\left(t\right)=0,\;\forall dr_{i}<dr_{i^{\prime }},\;i,i^{\prime }\in D \end{array}$$ (5)

$$\begin{array}{l} \overset{D}{\underset{i}{\sum }}q_{ij}\left(t\right)x_{ij}\left(t\right)=0,\;\forall sr_{j}>sr_{j^{\prime }},j,j^{\prime }\in S \end{array}$$ (6)

$$\begin{array}{l} requesttime_{i}\leq t,requesttime_{j}\leq t \end{array}$$ (7)

$$\begin{array}{lll} q_{ij}\left(t\right) & \geq & 0,q_{ij}\left(t\right)\in Z,i\in D,\;j\in S \end{array}$$ (8)

$$x_{ij}(t)=\{\begin{array}{l} 1,\text{ it is matched between }i\text{ and }j\\ 0,\text{ it is not matched between }i\text{ and }j \end{array}$$ (9)

The objective (1) of the model is to maximize the total quantity of resource matching. Constraint (2) ensures that each recipient *i* can obtain anti-epidemic resources that do not exceed their demand during the decision period *t*. Constraint (3) ensures that the supplier *j* can provide anti-epidemic resources without exceeding their maximum quantity. Constraint (4) indicates that the supplier *j* must provide resources to the recipient *i* within their service radius. Constraint (5) prioritizes the recipient *i*with a high level of epidemic prevention. Constraint (6) prioritizes suppliers *j* with a low supply level. Constraint (7) indicates that both the recipient *i* and the supplier *j* must arrive at the platform within the matching time window *t*. Constraint (8) ensures that the quantity of anti-epidemic resources allocated by suppliers to recipients is a non-negative integer. Constraint (9) indicates whether the supplier *j* allocates resources to the recipient *i*.

The MSODBM is a temporal and spatial dynamic decision-making problem characterized by unpredictability, major outbreaks, and limited information. The arrival of recipients and suppliers in the HIS is marked by uncertainty regarding both timing and quantity. The decision-making process for matching anti-epidemic resources is not only influenced by the current state of events but also has implications for the progression of future events. Given that the number of recipients and suppliers participating in the matching process, as well as the quantity of requested resources, varies across different matching time windows, the *average wait time* and the *average matching rate* serve as critical indicators for evaluating the outcomes of matching.

The total waiting time τ_*i*_ consists of waiting for the matching time $\tau_{i}^{batch}$ and the travel time $\tau_{i}^{travel}$. $\tau_{i}^{batch}$ refers to the difference between the match time and the request time for anti-epidemic resources. i.e., $\tau_{i}^{batch}=matchtime_{i}-requesttime_{i}$. $\tau_{i}^{batch}$ is influenced by the capacity of resource supply, the selection of time windows for platform matching, and the matching strategy employed. $\tau_{i}^{travel}$ represents the routing time following the allocation of anti-epidemic resources. Assuming a constant speed, $\tau_{i}^{travel}$ is determined by the distance between the supplier and the recipient.

The average wait time *avg*_τ_ is the ratio of the total waiting time for matching to the number of requests made by all recipients within the matching time window. It can be calculated by the Equation 10.


$$\begin{array}{l} avg_{\tau }\left(t\right)=\frac{\sum_{i\in M}\tau_{i}\left(t\right)}{\left|M\right|} \end{array}$$ (10)

The average match rate *avg*_*rate*_ is the ratio of the total completion rates of all recipients to the number of recipients within the specified matching time window. The average match rate can be calculated using Equation 11.


$$\begin{array}{l} avg_{rate}\left(t\right)=\frac{\sum_{i\in M}r_{i}\left(t\right)}{\left|M\right|} \end{array}$$ (11)

Where |***M***| indicates the total number of recipient *i* which participate in *t*; *r*_*i*_(*t*) indicates the matching completion rate of the recipient *i* which participate in *t*, as shown in Equation 12.


$$\begin{array}{l} r_{i}\left(t\right)=\frac{q_{matched}}{q_{i}} \end{array}$$ (12)

## Methods

### Matching time window selection

Choosing the appropriate matching time window significantly influences the waiting time experienced by resource recipients. There are two primary types of matching time windows: instant matching and batch matching (25). While instant matching can minimize individual waiting times, it may not always be the most optimal solution. Conversely, batch matching enhances the overall matching efficiency of the HIS platform (11). The size of the batch matching time window is also crucial. This study examines the *fixed time window batch matching strategy* (FTWBM) and the *adaptive time window batch matching strategy* (ATWBM).

In the FTWBM strategy, both the recipient and the supplier arrive randomly at the HIS platform over a time series. During a specified time period ***T***, the HIS platform divides this period into *n* matching time windows of equal duration (*t*_1_, *t*_2_, ⋯, *t*_*n*_). The size of each matching time window is *bs*. The HIS platform collects requests from recipients and suppliers within each matching time window and organizes resource matching at the conclusion of the window. For instance, if the time period is set to 24 h (*T* = 24h) and the matching time window is 10 min (*bs* = 10), a total of 144 fixed matching time windows can be generated. This implies that 144 resource matching tasks must be executed within a single day. Simultaneously, the HIS platform seeks to maximize the match within each time window.

Unlike the FTWBM, the ATWBM minimizes waiting time by automatically selecting a series of matching window sizes based on a specific strategy. The HIS platform also divides the time period ***T*** into *n* matching time windows (*t*_1_, *t*_2_, ⋯, *t*_*n*_), although the sizes of these windows may vary. Before each match begins, the HIS platform continues to gather requests from both recipients and suppliers to create a matching task set. Once the matching process is complete, the successfully matched recipients and suppliers are removed from their respective sets. In the subsequent matching time window, new requests from recipients and suppliers may arrive, or some recipients and suppliers may depart. Consequently, the recipient set ***D*** and the supplier set ***S*** are updated. The ATWBM dynamically selects an appropriate matching time window based on the feedback reward.

### Markov decision process for matching

The HIS platform utilizes batch matching, which allows resource suppliers and recipients to wait for a specified period. This process can be modeled as a Markov decision process. In this framework, the agent continuously interacts with the environment, receiving the current state from it at time *t*. Based on this state, the agent selects an action *A*, which then influences the environment, resulting in a reward *R*_*t*_ and a new state being returned to the agent. The Markov sequence stipulates that the generation of the next state depends solely on the current state. Specifically, when new suppliers or recipients arrive, two states—holding and matching—can be adopted. The action involves selecting a matching time window. Once the matching time window is determined, a matching timestamp *t* is generated, allowing the matching action to proceed. If *requesttime*_*i*_<*t* and the matching state is not a complete match (*rate*_*i*_ < 1), the recipient *i* is included in the recipient set ***D***_***t***_. If *requesttime*_*j*_<*t* and the matching state is not a complete match (*rate*_*j*_ < 1), the supplier *j* is included in the supplier set ***S***_***t***_. Those that do not meet the matching conditions will remain holding in the buffer (see Supplementary Figure S1). Meanwhile, the elements in the recipient and supplier sets are sorted according to time, respectively.

### Reinforcement learning-based adaptive time window batch matching

This study addresses the problem of Markov decision processes with batch matching by employing reinforcement learning to select adaptive time window sizes. Reinforcement learning involves mapping the relationship between learning states and behaviors, focusing on how to make decisions under uncertain conditions to maximize benefits (26). The multi-armed bandit (MAB) problem is a classic reinforcement learning challenge in which a system contains k arms, each yielding a different reward when selected. Decision-makers initially lack knowledge of the rewards associated with each action but gradually accumulate information through interactions with the system over a time period ***T***. The objective of the decision-maker is to maximize total revenue within ***T***. Exploration and exploitation are two fundamental concepts in reinforcement learning. Exploration refers to the strategy of trying various actions to discover the maximum reward, while exploitation involves selecting an action that is known to yield a substantial reward. The MAB framework gathers information by exploring different actions and uses it to maximize rewards through exploration-exploitation trade-offs. Additionally, the MAB is a key component of stochastic scheduling and is widely applied in fields such as online advertising, recommendation systems, and crowdsourcing tasks (27).

Adaptive time window batch matching addresses the mechanism of dividing matching time windows within a model to achieve the optimization goal of maximizing matches while minimizing waiting time. Since the rewards associated with specific matching time windows are not known in advance, the HIS platform interacts with the environment through exploration and exploitation strategies. Candidate matching sets are formed by selecting various batch matching time windows, after which the performance of the current state is observed, allowing for dynamic adjustments in subsequent selections. Through this process, the matching time window that yields the best performance is identified and selected to optimize the average waiting time. The MAB algorithm can be employed to solve these types of sequential decision-making problems. The division of matching time windows can be modeled as an MDP, where the current state and actions directly influence the next state. The primary challenge lies in selecting the appropriate matching window at the optimal time to maximize benefits. Equation 13 utilizes the average waiting time *R*_*t*_(*a*) of the matching time window *t* to calculate a score and update it accordingly.


$$\begin{array}{l} R_{t}\left(a\right)=\frac{a_{i1}+a_{i2}+\cdots +a_{ic}}{c} \end{array}$$ (13)

Where *a*_*ic*_ indicates the average waiting time of the matching time window *t* for the *c*-th selection; *c* indicates the total number of times the matching time window *t* has been selected.

This study selects the matching time window based on the classic ε-greedy algorithm (26). The sizes of the matching time window set *A* = {*bs*_1_, *bs*_2_, ⋯, *bs*_*k*_} are utilized as the k-arms of MAB. As illustrated in Algorithm 1, a matching time window *t*_*i*_ is randomly selected with the probability of ε(0 < ε < 1) to explore, while the time window with the highest current reward is chosen to exploit with a probability of 1−ε. Whenever the matching of *t*_*i*_ ends, the revenue of *t*_*i*_ is updated. To facilitate the rapid learning of the online version, this study introduces a continuous policy improvement interval *K*_θ_ and the decay coefficient λ ∈ (0, 1) of ε_*k*_. When *K*_θ_ = 0, each time the online algorithm performs a match, the matching time window is updated to initiate a new round of matching, which is entirely optimistic (28). When *K*_θ_>1, the algorithm executes several matches using the current strategy before refining it, which is considered partially optimistic. Furthermore, as the number of matches increases, ε_*k*_ typically decreases. The algorithm will employ the current strategy with a higher probability (29). Consequently, Equation 14 establishes the initial exploration probability ε_0_, and each match decays exponentially according to the decay rate λ.


$$\begin{array}{l} \varepsilon_{k}=\varepsilon_{0}\lambda^{k} \end{array}$$ (14)

Algorithm 1 Adaptive time window batch bipartite matching algorithm.

**Input:** the sizes of time window batch set ***BS***, the timestamp set ***TS***, policy improvement interval, the exploration coefficient, the decay coefficient λ.
**Output:**
The maximum matching set ***M***, the average waiting time set ***AT***, and the average matching rate ***RT***.
**Algorithm**
1: *R*←[0for*bs*in***A***] 
2: ω←0, initialize time window batch *bs*_0_ 
3: for *k* = 1, 2, ⋯  do: 
4: if *random*(0, 1) ≤ ε_*k*_ then 
5: *bs*←*random choice*(*BS*) 
6: else 
7: *bs*←*bs*_ω_ 
8: end if 
9: set the match timestamp as *ts* 
10: get recipients set ***D*** and suppliers set ***S*** smaller than the current timestamp and the matching status < 1 respectively 
11: do Algorithm 2 
12: calculate*R*_*t*_ 
13: *R*[*bs*]←*R*_*t*_ 
14: if *k* = =(ω+1)*K*_θ_ then 
15: *bs*_ω+1_←*A*[min(*R*)] 
16: ω←ω+1 
17: end if 
18: end for

### Nearest neighbor first-based bipartite matching

The supply-demand bipartite matching network is based on the service radius of suppliers. Equation 4 demonstrates that each supplier *j* is constrained by their service scope, with the maximum service scope being defined as *l*_*j*_. In the bipartite network *G*<*U, V, E*(*U, V*)> consisting of suppliers and recipients, where *U* = ***D***_*t*_, and *V* = ***S***_*t*_, calculate Manhattan distance *l*_*ij*_ between the recipient *i* and the supplier *j*. According to the Equation 4, if *l*_*ij*_ ≤ *l*_*j*_, there is an edge between *i* and *j*, and the distance is used as the weight of the edge, that is, *E*(*i, j*) = *l*_*ij*_. if *l*_*ij*_>*l*_*j*_, there is no match between *i* and *j*, and set *E*(*i, j*) = −1.

The nearest neighbor first (NNF) strategy considers the distance between suppliers and recipients by incorporating distance-based priority matching constraints. Equation 15 prioritizes the allocation of anti-epidemic resources from suppliers to recipients that are closer in proximity, thereby maximizing matching while minimizing $\tau_{i}^{travel}$. Let *L*_*i*_ represent a set of *l*_*ij*_, which includes all suppliers that can be matched with recipients. Select min(*l*_*ij*_) as the prioritized matching for recipients (see Supplementary Figure S2).


$$\begin{array}{l} \overset{D}{\underset{i}{\sum }}x_{ij}\left(t\right)q_{ij}\left(t\right)=0,\;\forall l_{ij^{\prime }}<l_{ij} \end{array}$$ (15)

Multi-stage bipartite graph matching is predicated on the priorities of suppliers and recipients. Based on the recipient's anti-epidemic priority attribute *dr* and Equation 5, the bipartite network *G*<*U, V, E*(*U, V*)> is partitioned into *G*_*PH*_<*U, V, E*(*U, V*)> with high-priority recipients, and *G*_*PL*_<*U, V, E*(*U, V*)>with low-priority recipients (see Supplementary Figure S3). Subsequently, *G*_*PH*_<*U, V, E*(*U, V*)> and *G*_*PL*_<*U, V, E*(*U, V*)> are divided into three stages according to the supplier's level *sr* = { 1, 2, 3 } , respectively (see Supplementary Figure S4). As shown in Algorithm 2, the set ***U*** matches the set ***V*** with *sr* = 1 in the first stage. If the matching is not fully completed, the second stage of matching is performed, wherein set ***U*** matches set ***V*** with *sr* = 2. Similarly, the third stage of matching is conducted until the matching is successfully completed. If, at any stage, set ***U*** completes matching, the process is terminated.

Algorithm 2 Service-level-based multi-stage matching algorithm (G-HMBMC).

**Input:**
G < U,V,E(U,V)>
**Algorithm**
1: *SR* = {1, 2, 3} 
2: for *sr* ∈ *SR* do: 
3: *V*_*sr*_←*j*, for *j* in *V* and *j*_*sr*_ = *sr* 
4: if *G*_*PH*_<*U, V, E*(*U, V*)>then 
5: do GMBMC algorithm 
6: else 
7: do HMBMC algorithm 
8: end if 
9: if *U* = =∅ then 
10: break 
11: end if 
12: end for

Prioritizing the allocation of resources to areas that have been significantly impacted by the epidemic can aid in containing outbreaks within these areas and in preventing further transmission. In the process of matching anti-epidemic resources, recipients with higher priority levels are given precedence in the matching procedure. The Greedy-based many-to-many bipartite matching algorithm with capacity (see Algorithm S1) operates under the assumption that the current high-priority recipient will consistently be matched with the most advantageous supplier, irrespective of the matching decisions made by other recipients.

For the set of recipients with low priority, the maximum matching is determined using the classic Hungarian algorithm, a combinatorial optimization algorithm for solving task allocation problems in polynomial time. This algorithm identifies the maximum matching by continuously locating augmenting paths to increase the number of matching edges and vertices. It effectively addresses task allocation challenges to optimize resource utilization and is widely applied across various fields involving resource matching problems. The classic Hungarian algorithm primarily addresses assignments involving a single task or resource, without accounting for capacity constraints. Conversely, the allocation of anti-epidemic resources must consider the quantity of resources allocated, presenting a matching problem with capacity limitations. Therefore, this study incorporates capacity attributes into the vertices of bipartite graph networks (see Algorithm S2) and defines a recursive function *getPath*(*i, q*_*i*_) to identify augmenting paths. Additionally, the classic Hungarian algorithm is designed to solve one-to-one matching problems. However, there exists a mismatch of anti-epidemic resources between recipients and suppliers, resulting in a many-to-many matching problem. Consequently, it is essential to extend the classic Hungarian algorithm to accommodate many-to-many matching, which can be achieved by enhancing the augmenting path search mechanism.

## Experimental analysis

### Experimental setup

This study delineates the findings of an experiment conducted utilizing the proposed algorithm on synthetic datasets. The decision period is established at |***T***| = 120. Within this timeframe, recipient requests and supplier resources are introduced randomly in chronological order, generating corresponding data based on the characteristics of both resource recipients and suppliers. The anti-epidemic level (*dr* = {*high, low*}) of the recipients is randomly assigned in accordance with the demand response, while the suppliers' levels (*sr* = {1, 2, 3}) are generated randomly based on the supply response. In consideration of the availability of anti-epidemic resources, the ratio of the quantity of resources requested by the recipient to those supplied by the supplier is approximately set at 0.8. A total of 3,000 sets of recipient requests and 1,800 sets of supplier resources are generated randomly.

The FTWBM experiment selects any matching time window from the ***BS*** for testing, while the ATWBM experiment randomly selects a matching time window *t* with a probability of ε_*k*_ for exploration. The remaining time window is then selected for exploitation with a probability of 1−ε_*k*_. To facilitate rapid learning in the online version, Table 1 establishes the decay coefficient λ for ε_*k*_ and the continuous strategy improvement intervals *K*_θ_.

**Table 1:** Experimental parameter settings.

**Parameters**	**Values**
|***T***|	120
|***D***|	3,000
|***S***|	1,800
BS	{1,2,3,4,6,8}
ε_0_	{0.02, 0.2,0.8}
*K*_θ_	{0,2}
λ	{0.2,0.8}

### Experimental results

#### Impact of fixed batch size on the matching time window

**Figure 2:**
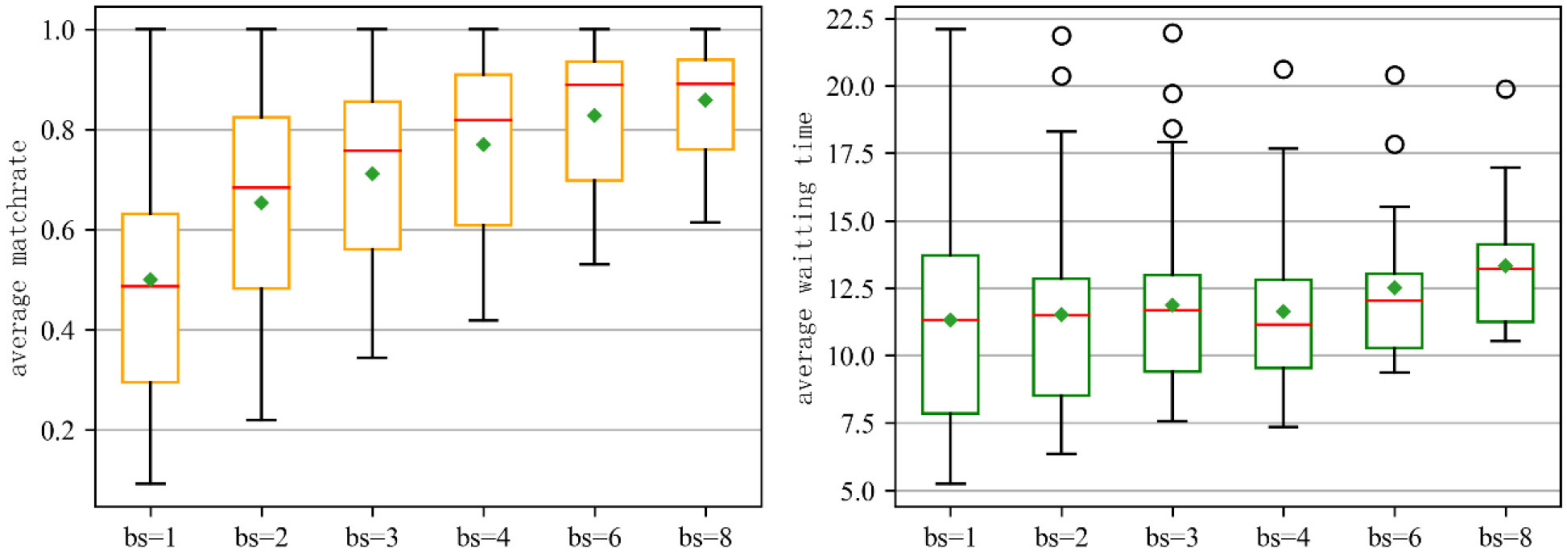
Results across batch sizes (bs). **Left**: Average match rate; **Right**: Average waiting time. Each boxplot shows median (red line), interquartile range (box), whiskers (1.5 × IQR), outliers (circles), and mean (green diamond).

Figure 2 illustrates the average matching rate and average waiting time for anti-epidemic resources under fixed matching time windows with varying batch sizes. As batch size increases from 1 to 8, the average match rate shows a clear upward trend, with median values rising from approximately 0.5 to 0.89, indicating improved matching efficiency at larger batch sizes. The instant matching result (*bs* = 1) is inferior to the delayed batch matching across other fixed time windows in terms of average matching rate. Conversely, the average waiting time initially decreases, then rises after exceeding a certain threshold. The average waiting time gradually decreases from *bs* = 1 to *bs* = 4, after which it begins to rise (from *bs* = 4 to *bs* = 8). Indeed, *bs* = 4 strikes a balance between average waiting time and matching rate, making it an optimal choice for a fixed batch matching time window. It facilitates a reasonable waiting time while achieving a satisfactory matching rate. The result demonstrates that increasing batch size enhances matching efficiency but risks increasing waiting time variability. While larger batches yield higher match rates, they do not consistently reduce waiting time and may even worsen it due to aggregation delays. Therefore, batch size should be carefully tuned to achieve the best balance between matching performance and user experience. It is particularly important to dynamically adjust the batch size through an adaptive batching strategy.

From a statistical perspective, Table 2 reveals a clear and statistically significant improvement in average match rate (AR) with increasing batch size (*bs*), as evidenced by non-overlapping 95% confidence intervals across all conditions, indicating consistent and reliable gains. Conversely, while average waiting time (AT) increases monotonically, the substantial overlap between 95% CIs suggests that most pairwise differences are not statistically significant. This highlights a trade-off where higher matching efficiency comes at the cost of longer delays, but only marginally so until larger batches. Notably, the precision of AR estimates remains high due to narrow CIs and low standard errors, whereas AT estimates become less reliable as sample sizes decrease, reflected in wider CIs. Overall, the data support a strong positive effect of batch size on matching performance with high statistical credibility, while the impact on waiting time is less certain. *bs* = 4 emerges as an optimal balance, achieving high AR (77.0%, 95% CI [0.706, 0.834]) with minimal delay increase (11.64 min, 95% CI [10.501, 12.777]) and sufficient statistical precision.

**Table 2:** Statistical results of average match rate and average waiting time across different batch sizes (*bs*).

	**The average match rate (AR)**						**The average waiting time (AT)**					
	***bs*** = **1**	***bs*** = **2**	***bs*** = **3**	***bs*** = **4**	***bs*** = **6**	***bs*** = **8**	***bs*** = **1**	***bs*** = **2**	***bs*** = **3**	***bs*** = **4**	***bs*** = **6**	***bs*** = **8**
n	120	60	40	30	20	15	120	60	40	30	20	15
*median*	0.486	0.684	0.757	0.819	0.889	0.890	11.325	11.507	11.675	11.153	12.039	13.213
* $\bar{x}$ *	0.500	0.654	0.711	0.770	0.828	0.859	11.310	11.516	11.862	11.639	12.520	13.330
*s*	0.248	0.220	0.195	0.172	0.145	0.119	3.803	3.411	3.344	3.048	2.830	2.616
SE	0.023	0.028	0.031	0.031	0.033	0.031	0.347	0.440	0.529	0.556	0.633	0.675
ME	0.045	0.057	0.062	0.064	0.068	0.066	0.687	0.881	1.069	1.138	1.325	1.448
95% CI	[0.456, 0.545]	[0.597, 0.711]	[0.649, 0.773]	[0.706, 0.834]	[0.760, 0.896]	[0.793, 0.925]	[10.622, 11.997]	[10.635, 12.397]	[10.792, 12.931]	[10.501, 12.777]	[11.196, 13.845]	[11.882, 14.779]

#### Impact of adaptive batch sizes on the matching time window

In the context of online matching for anti-epidemic resources utilizing the ATWBM strategy, this study enhances the effectiveness of the G-HMBMC algorithm by dynamically selecting the matching time window through control parameters (see Supplementary Figure S5): initial exploration probability ε_0_, strategy improvement interval *K*_θ_ and attenuation coefficient λ of ε_*k*_. The subsequent sections discuss the impact of these parameters on the matching results.

**Figure 3:**
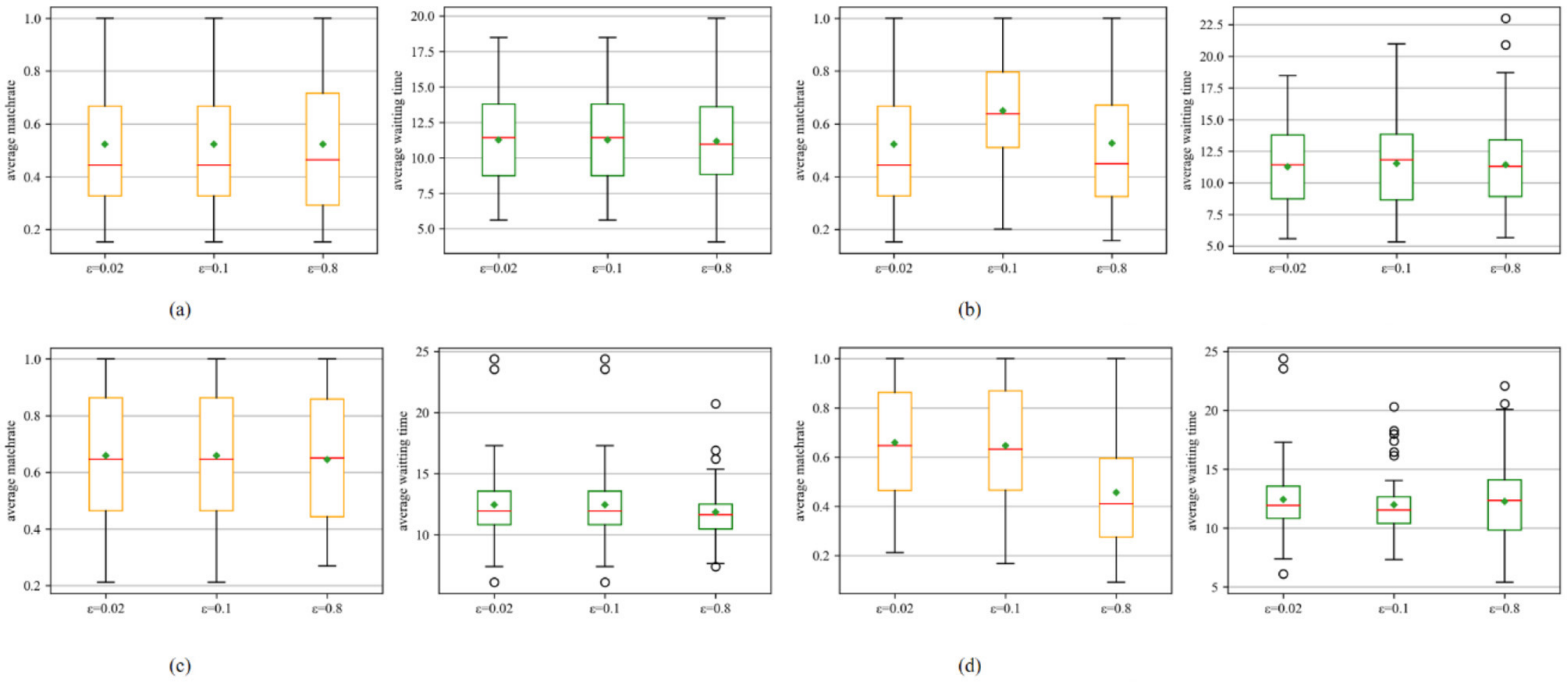
Impact of the initial exploration probability ε_0_ on the matching result. Each boxplot shows median (red line), interquartile range (box), whiskers (1.5 × IQR), outliers (circles), and mean (green diamond). **(a)** Result on self-adaptive batch size (*K* = 0, λ = 0.2). **(b)** Result on self-adaptive batch size (*K* = 0, λ = 0.8). **(c)** Result on self-adaptive batch size (*K* = 2, λ = 0.2). **(d)** Result on self-adaptive batch size (*K* = 2, λ = 0.8).

Figure 3 illustrates the effect of the attenuation coefficient λ on the matching results. The data indicate that, in most instances, the matching results under partially optimistic scenarios are superior to those observed in fully optimistic scenarios. In the fully optimistic scenario with *K*_θ_ = 0, when the attenuation coefficient is low (λ = 0.2), a larger initial exploration probability (ε_0_ = 0.8) can yield improved matching results, as shown in Figure 3a. Conversely, when a larger attenuation coefficient (λ = 0.8) is employed, a smaller initial exploration probability (ε_0_ = 0.1,ε_0_ = 0.02) can yield improved matching results, as shown in Figure 3b. In the scenario of partial optimism with *K*_θ_ = 2, when the attenuation coefficient is low (λ = 0.2), the selection of the attenuation coefficient (ε_0_) does not significantly influence the matching outcomes, as demonstrated in Figure 3c. However, when the attenuation coefficient is high (λ = 0.8), a smaller initial exploration probability (ε_0_ = 0.1,ε_0_ = 0.02) facilitates the exploration of better matching time windows, leading to improved matching results, as shown in Figure 3d. The result indicates that with a low attenuation coefficient, the algorithm can maintain a larger exploration space to identify better matching strategies. Conversely, with a low initial exploration probability, the algorithm tends to prioritize the current optimal strategy and neglect exploration, potentially missing the opportunity to discover the optimal matching strategy. In particular, the application of a fully optimistic strategy with a small attenuation coefficient and a partially optimistic strategy with a small attenuation coefficient both produce favorable matching results. These findings underscore the critical importance of maintaining an appropriate level of exploration to achieve improved matching outcomes.

### Discussion

#### Comparison between FTWBM and ATWBM

In the FTWBM strategy, a limited batch size within the matching time window constrains the available matching opportunities for both suppliers and recipients. This restriction leads to suboptimal resource matching and diminished matching rates, consequently increasing the average waiting time. The restricted time window allows only a limited number of supply and demand pairs to be successfully matched, requiring other resources to await the subsequent time window for potential matching. In this context, while the waiting time may be brief, the overall matching rate remains low. Conversely, when the batch size of the matching time window is excessively large, the number of participating supply and demand pairs escalates significantly. This increase can improve the matching rate for both parties, as more resources are allowed to find appropriate matches. However, this approach also complicates the matching process, as the system must handle a larger volume of matching requests. Consequently, this may result in longer online waiting times for recipients, as the matching process may require additional time to identify suitable matches. Therefore, this study aims to determine a relatively optimal matching time window that effectively balances the matching rate and average waiting time through experimental analysis. Nonetheless, in contexts where the progression of the epidemic is unpredictable, and both suppliers and recipients experience dynamic changes, this optimal point may not remain constant. Furthermore, this study corroborates that instant matching is generally less effective in terms of matching outcomes when compared to delayed batch matching.

In comparison to the FTWBM strategy, the optimal performance in partially optimistic scenarios is generally similar to that observed with *bs* = 3. In fully optimistic scenarios, the optimal performance also closely resembles that associated with *bs* = 2. The optimal window size in the FTWBM strategy outperforms the results of the ATWBM strategy. However, the ATWBM strategy demonstrates greater effectiveness in adapting to the dynamic changes in epidemic prevention and control, particularly in partially optimistic scenarios. By optimizing the control parameters of the adaptive time window algorithm, the matching performance can be continuously enhanced. In terms of optimizing matching outcomes, the ATWBM strategy holds a more significant advantage over the FTWBM strategy.

#### Comparison of different MBA algorithms

**Figure 4:**
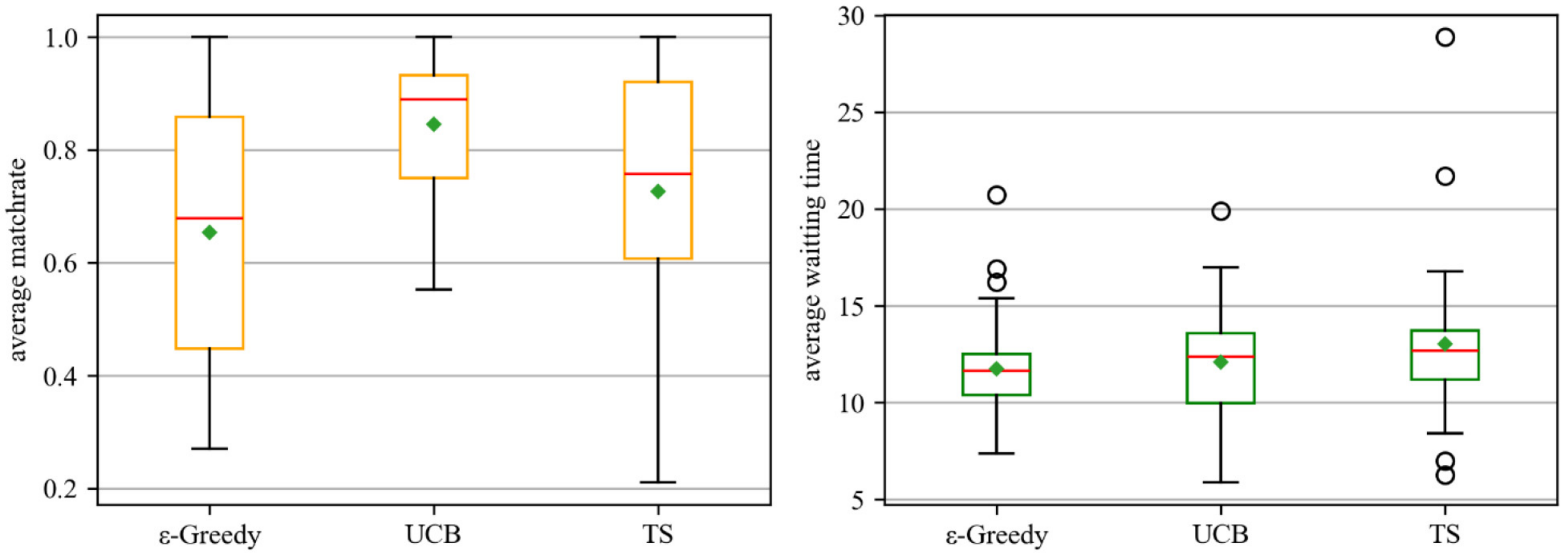
Results of ε-Greedy, UCB, and Thompson Sampling. Each boxplot shows median (red line), interquartile range (box), whiskers (1.5 × IQR), outliers (circles), and mean (green diamond).

This study compares the performance differences among Upper Confidence Bound (UCB), Thompson Sampling (TS) and ε-Greedy and calculate the corresponding cumulative regret of the algorithm. According to Figure 4, the two box plots compare the performance of three reinforcement learning algorithms across two key metrics: average match rate (depicted in the left plot) and average waiting time (shown in the right plot). In the left plot, UCB demonstrates the most favorable performance. It features the highest median (indicated by the red line) and mean (marked by the green diamond), along with a relatively compact box that signifies a narrow interquartile range, implying consistent and high match rates. Conversely, ε-Greedy exhibits a wider box and a lower median, with whiskers extending to both low and high extremes, reflecting greater variability in its match rate. TS falls in between: its median match rate is positioned between those of ε-Greedy and UCB, yet it includes outliers with exceptionally low match rates, indicating less stability than UCB. In the right plot, ε-Greedy shows the lowest median and mean waiting times. However, it also contains outliers with extremely high waiting times, suggesting that while it typically has short waiting periods, there are instances of significant delays in the process. The UCB's box is centered at a moderate level, indicating moderately stable waiting times without extreme outliers. TS has a higher median and mean waiting time than the other two algorithms. Its whiskers extend to both very low and very high values, indicating that its waiting time is less stable and more variable than that of both ε-Greedy and UCB. In summary, UCB stands out for its excellent and stable average matching rate, making it a leader in terms of successful matching consistency. ε-Greedy performs the shortest average waiting time but suffers from greater variability.

Table 3 compares three reinforcement learning strategies—ε-Greedy, Thompson Sampling (TS), and Upper Confidence Bound (UCB)—highlighting significant differences in matching performance, as quantified by the average match rate (AR), while showing no statistically significant differences in average waiting time (AT). For AR, UCB achieves the highest mean (0.845) with a narrow 95% confidence interval [0.780, 0.910], which does not overlap with that of ε-Greedy ([0.575, 0.730]), indicating a highly significant improvement. TS performs moderately (0.726, [0.636, 0.815]) and shows partial overlap with both ε-Greedy and UCB, suggesting no clear advantage over ε-Greedy but possibly inferior to UCB. Conversely, for AT, all three methods show overlapping 95% CIs—ranging from [10.287, 13.849] (UCB) to [11.348, 14.724] (TS)—indicating no statistically significant differences in delay. Notably, UCB and TS have much wider CIs than ε-Greedy due to smaller sample sizes and higher variability, reducing their reliability. Overall, UCB emerges as the most effective strategy, offering significantly higher match rates without demonstrably increasing waiting time, and this is supported by high statistical precision. ε-Greedy provides shorter delays with reliable estimates but at the cost of lower matching efficiency.

**Table 3:** Statistical results of average match rate and average waiting time for ε-Greedy, UCB, and Thompson Sampling.

	**The average match rate (AR)**			**The average waiting time(AT)**		
	ε**-Greedy**	**Thompson Sampling (TS)**	**Upper Confidence Bound (UCB)**	ε**-Greedy**	**Thompson Sampling (TS)**	**Upper Confidence Bound (UCB)**
n	38	28	18	38	28	18
*median*	0.678	0.757	0.889	11.625	12.679	12.373
$\bar{x}$	0.653	0.726	0.845	11.740	13.036	12.068
*s*	0.236	0.232	0.131	2.731	4.353	3.581
SE	0.038	0.044	0.031	0.443	0.823	0.844
ME	0.078	0.090	0.065	0.898	1.688	1.781
95% CI	[0.575, 0.730]	[0.636, 0.815]	[0.780, 0.910]	[10.842, 12.638]	[11.348, 14.724]	[10.287, 13.849]
*CIW*	0.155	0.179	0.13	1.796	3.376	3.562

#### Ethics and fairness

The core nature of anti-epidemic resource allocation lies in its public health emergency attribute—that is, resource allocation must first serve the collective goal of rapidly controlling the spread of the epidemic and reducing the overall risk of infection or death. This premise defines the trade-off principle, which states that group fairness takes priority over absolute individual fairness. The adaptive time window selection and G-HMBMC multi-stage matching algorithm prioritize ensuring that high-risk groups have access to anti-epidemic resources, which aligns with the emergency attribute of anti-epidemic resource allocation. Meanwhile, by selecting a matching time window with the minimum average waiting time, the algorithm compensates for the potential individual unfairness that may result from prioritizing the group. Through differentiated adaptation in the time dimension and hierarchical regulation during the matching process, the algorithm safeguards the core goal of group fairness. It avoids systematic unfairness at the individual level. Ultimately, it achieves synergy between public health efficiency and fairness.

#### Complexity analysis

The proposed online bandit algorithms (greedy, TS, or UCB) for a dynamic matching problem jointly optimize the matching configuration and arm selection in a sequential decision loop. The overall time complexity is predominantly determined by the main experimental loop and the graph matching algorithms employed within it, rather than by the bandit selection logic itself. The core bandit algorithms, whether Thompson Sampling or UCB, are highly efficient. With a fixed number of arms (K = 6), operations such as choosing an arm and updating the reward estimates are performed in constant time, O(K) = O(1), per time step. The primary computational bottleneck arises from the matching operations (Greedy or Hungarian) required for each selected arm in every iteration. The worst-case time complexity is governed by the Hungarian algorithm, which has a complexity of O(max(N, M)^∧^3) for bipartite graphs with sets of sizes N and M. This operation is executed a fixed number of times (e.g., six times for different data subsets) per time step T. Consequently, the overall worst-case time complexity is O(T^*^ max(N, M)^∧^3). If a greedy matching algorithm is used instead, this could be reduced to approximately O(T^*^N^*^M). The loop count T is not a direct input variable but is determined by the dynamics of the external environment; however, it contributes a linear factor to the total running time. Therefore, the scalability of the algorithm is highly sensitive to the sizes of the sets being matched (N and M). Regarding space complexity, the requirements are driven by the data structures required for the matching process. Bandit algorithms are memory-efficient and require only O(K) space to store parameters. Significant memory consumption occurs within the experimental loop. The algorithm must store the sets of bipartite graphs, which requires O(N+M) space. Furthermore, matching algorithms, particularly the Hungarian algorithm, often require an O(N^*^M) distance matrix or similar data structures to operate. The bandit list, which records the estimated values for all arms at each time step, consumes O(T^*^K) space, which is typically dominated by the O(N^*^M) requirement for the matching matrices when N and M are large. Thus, the overall space complexity is O(N^*^M). In practice, for large graphs, the O(N^*^M) term is the dominant memory constraint.

## Conclusion

The primary contributions and managerial insights of this study are as follows. First, this study showed that delayed batch matching generally produces superior overall matching results than instant matching in the context of online matching of anti-epidemic resources. As the matching time window expands, the average waiting time initially decreases, then rises again, while the average matching rate continues to increase to a certain extent. Therefore, healthcare operations managers should strive to find a balance between average waiting time and average matching rate when selecting a matching time window, prioritizing a window that offers low average waiting time and high average matching rate. Secondly, there is no universally optimal or static matching time window in the context of unpredictable epidemic developments and dynamic fluctuations in supply and demand. Healthcare operations managers should periodically adjust the matching time window based on trends in epidemic progression and resource availability. In scenarios where the epidemic spreads rapidly, resulting in increased resource requests from the infected demand side and potential supply shortages, a larger matching time window can increase the average matching rate; however, this may lead to longer waiting times. Conversely, a smaller matching time window may also be effectively utilized. Thirdly, experiments conducted on the proposed adaptive time window matching strategy indicate that a higher exploration probability significantly improves resource matching efficiency. Given the uncertain supply and demand conditions during an epidemic, maintaining a high level of exploration within the matching time window is crucial for effective resource allocation. Additionally, in practical applications, health operations managers also need to determine accurate travel time in conjunction with navigation maps.

While the model presented effectively addresses the online matching problem of anti-epidemic resources, certain limitations necessitate further investigation. This study focuses solely on the impact of temporal factors on the allocation of epidemic prevention and control resources. However, other critical factors, such as resource supply capacity, the cross-regional distribution of resource shortages, population concentration, and spatial distribution, also require substantial research attention. Simultaneously, the issue of balancing efficiency and fairness in epidemic resource allocation is equally worthy of research consideration.

## Data Availability

The raw data supporting the conclusions of this article will be made available by the authors, without undue reservation.

## References

[1] Ma J Xu J Zhao XL Huo SL Duan XL Mu YS . Several major issues concerning the environmental transmission and risk prevention of SARS-CoV-2. Science China-Earth Sciences. (2022) 65:1047–56. doi: 10.1007/s11430-021-9918-9 35578665 PMC9097562

[2] Fischer GS Righi RDR Ramos GDO da Costa C Rodrigues JJPC. ElHealth: Using Internet of Things and data prediction for elastic management of human resources in smart hospitals. Eng Appl Artif Intell. (2020) 87:103285. doi: 10.1016/j.engappai.2019.103285

[3] Shapley L Scarf H. On cores and indivisibility. J Mathem Econ. (1974) 1:23–37. doi: 10.1016/0304-4068(74)90033-0

[4] Shapley LS Shubik M. The assignment game I: The core. Int J Game Theory. (1971) 1:111–30. doi: 10.1007/BF01753437

[5] Roth AE. Incentive compatibility in a market with indivisible goods. Econ Lett. (1982) 9:127–32. doi: 10.1016/0165-1765(82)90003-9

[6] Roth AE. Marketplaces, markets, and market design. Am Econ Rev. (2018) 108:1609–58. doi: 10.1257/aer.108.7.1609 30091861

[7] Roth AE. The college admissions problem is not equivalent to the marriage problem. J Econ Theory. (1985) 36:277–88. doi: 10.1016/0022-0531(85)90106-1

[8] Huang Z Tang ZG Wu X Zhang Y. Online vertex-weighted bipartite matching: beating 1-1/e with random arrivals. ACM Trans Algorithms. (2019) 15:38. doi: 10.1145/3326169

[9] Torrico A Toriello A. Dynamic relaxations for online bipartite matching. Informs J Comput. (2022) 34:1871–84. doi: 10.1287/ijoc.2022.1168

[10] Fahrbach M Zadimoghaddam M Huang Z Tao R. Edge-weighted online bipartite matching. J ACM. (2022) 69:45. doi: 10.1145/3556971

[11] Chen W Zheng J Yu H Chen G Chen Y Li D. Online learning bipartite matching with non-stationary distributions. ACM Trans Knowl Discov Data. (2022) 16:83. doi: 10.1145/3502734

[12] Goyal V Udwani R. Online matching with stochastic rewards: optimal competitive ratio via path-based formulation. Oper Res. (2023) 71:563–80. doi: 10.1287/opre.2022.2345

[13] Bai G Velamuri SR. Contextualizing the sharing economy. J Managem Stud. (2021) 58:977–1001. doi: 10.1111/joms.12652

[14] Slee T. What's Yours Is Mine: Against the Sharing Economy, New York, NY: OR Books. (2017).

[15] Li T. Trans-regional medical support in public health emergencies: a case study of Wuhan in the early COVID-19 pandemic in China. Risk Manag Healthc Policy. (2022) 15:677–83. doi: 10.2147/RMHP.S346556 35449543 PMC9017699

[16] Nowzari C Preciado VM Pappas GJ. Analysis and control of epidemics: a survey of spreading processes on complex networks. IEEE Control Syst Magaz. (2016) 36:26–46. doi: 10.1109/MCS.2015.2495000

[17] Chen Z-Y Sun M Han X-X. Prediction-driven collaborative emergency medical resource allocation with deep learning and optimization. J Operat Res Soc. (2022) 74:1–14. doi: 10.1080/01605682.2022.2101953

[18] Pan Y Cheng TCE He Y Ng CT Sethi SP. Foresighted medical resources allocation during an epidemic outbreak. Transport Res Part E: Logist Transport Rev. (2022) 164:102762. doi: 10.1016/j.tre.2022.102762

[19] Long EF Nohdurft E Spinler S. Spatial resource allocation for emerging epidemics: a comparison of greedy, myopic, and dynamic policies. Manufact Serv Operat Managem. (2018) 20:181–98. doi: 10.1287/msom.2017.0681

[20] Pei Z Yuan Y Yu T Li N. Dynamic allocation of medical resources during the outbreak of epidemics. IEEE Trans Automat Sci Eng. (2022) 19:663–76. doi: 10.1109/TASE.2021.3102491

[21] Zhu X Liu Y Wang S Wang R Chen X Wang W. Allocating resources for epidemic spreading on metapopulation networks. Appl Math Comput. (2021) 411:126531. doi: 10.1016/j.amc.2021.126531

[22] Enayati S Özaltin OY. Optimal influenza vaccine distribution with equity. Eur J Oper Res. (2020) 283:714–25. doi: 10.1016/j.ejor.2019.11.025

[23] Chen XW Chong WF Feng RH Zhang LF. Pandemic risk management: resources contingency planning and allocation. Insurance Mathem Econ. (2021) 101:359–83. doi: 10.1016/j.insmatheco.2021.08.001 34803199 PMC8593845

[24] Gatouillat A Badr Y Massot B Sejdić E. Internet of medical things: a review of recent contributions dealing with cyber-physical systems in medicine. IEEE Intern Things J. (2018) 5:3810–22. doi: 10.1109/JIOT.2018.2849014

[25] Ashlagi I Nikzad A Strack P. Matching in dynamic imbalanced markets. Rev Econ Stud. (2022) 90:1084–24. doi: 10.1093/restud/rdac044

[26] Sutton RS Barto AG. Reinforcement Learning: An Introduction. Cambridge, MA: The MIT Press. (2018).

[27] Bouneffouf D Rish I. A survey on practical applications of multi-armed and contextual bandits. arXiv [preprint] arXiv:1904.10040. (2019). doi: 10.48550/arXiv.1904.10040

[28] Sutton RS. Learning to predict by the methods of temporal differences. Mach Learn. (1988) 3:9–44. doi: 10.1007/BF00115009

[29] Li L Littman ML Mansley CR. Online exploration in least-squares policy iteration. In: AAMAS '09. Richland, SC: International Foundation for Autonomous Agents and Multiagent Systems (2009). p. 733–9.

