# Supplementary material for: Online bipartite matching methodology for anti-epidemic resources allocation: an adaptive time window based on reinforcement learning
Source: Front Public Health. 2026 Jan 8;13:1644499. doi: 10.3389/fpubh.2025.1644499 (PMC12823865; doi:10.3389/fpubh.2025.1644499)
Supplement: Supplementary file 1 [file Data_Sheet_1.docx]

Supplementary Material

# Supplementary Figures

**Supplementary Figure 1**


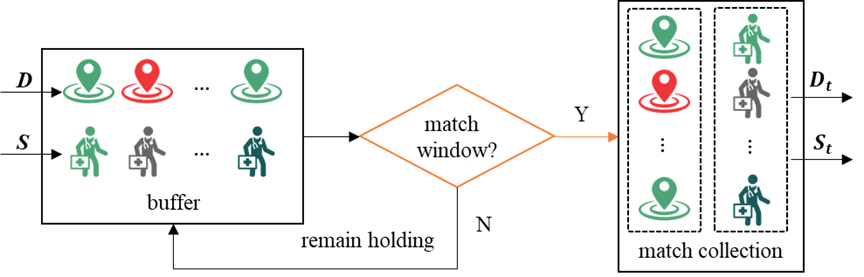


**Figure S1. Matching set of suppliers and demanders in matching time window**

**Supplementary Figure 2**


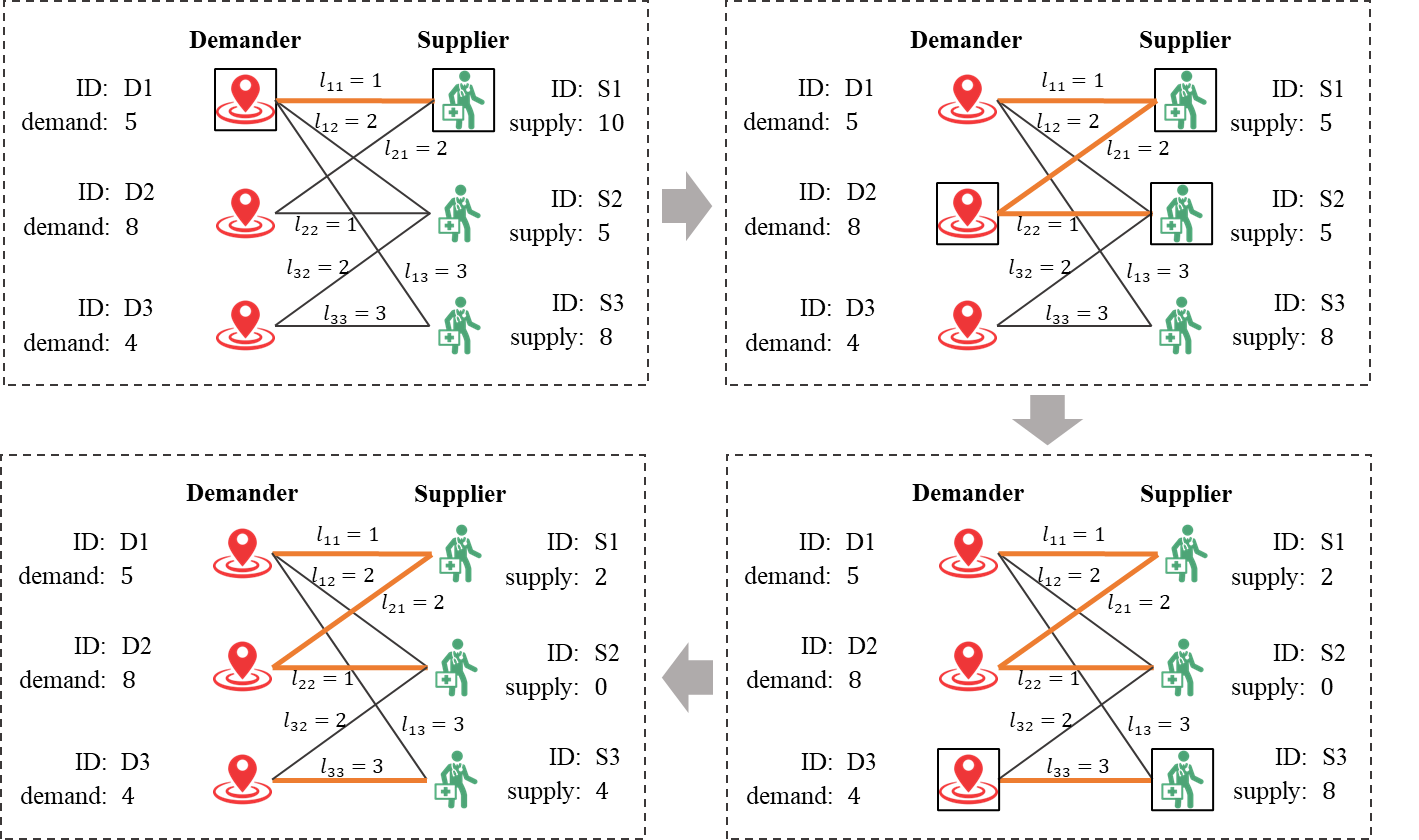


**Figure S2.** An example matching process of MSODBM

**Figure S2** shows a matching example of $t=0$. $l_{11}$ is the minimum value in $L_{1}=\{l_{11},l_{12},l_{13}\}$ for the demander D1. This shows that the Manhattan Distance between D1 and S1 is the closest. S1 is preferentially matched to D1 with 5 units match quantity. The matching set of D1 is $\text{(D1,S1,5,}t_{0}\text{,1)}$. For D2, $L_{2}=\{l_{22},l_{21}\}$, firstly match S2 to D2 with 5 units match quantity, and then match S1 to D2 with 3 units match quantity. The matching set of D2 is $\text{(D2,S2,5,}t_{0}\text{,}\frac{5}{8}\text{)}$ and $\text{(D2,S1,3,}t_{0}\text{,}\frac{3}{8}\text{)}$. Similarly, the matching set of D3 is $\text{(D3,S3,4,}t_{0}\text{,1)}$. The total travel time is 3 and the average time is 2.

**Supplementary Figure 3**


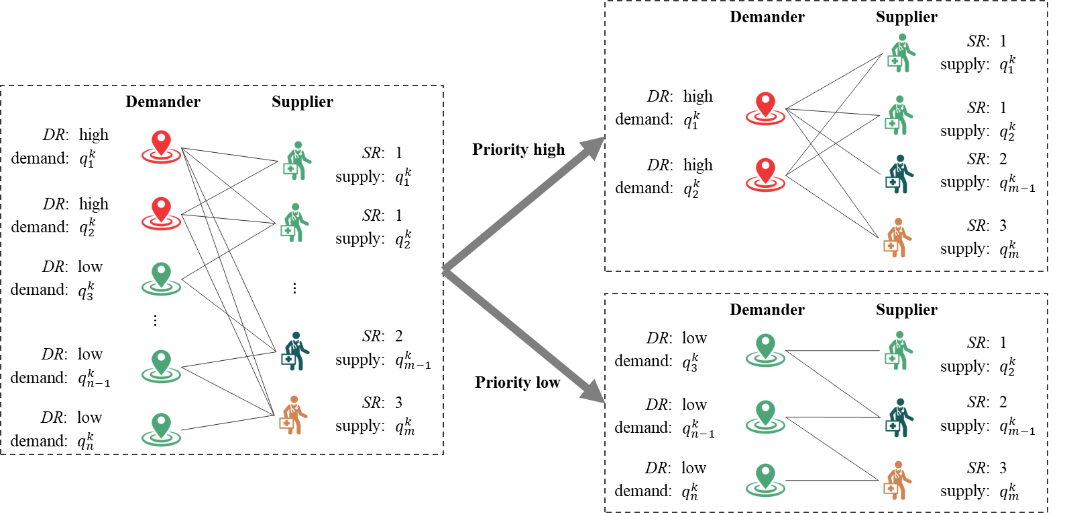


**Figure S3.** Divided the bipartite graph network according to demander’s priority of anti-epidemic

**Supplementary Figure 4**


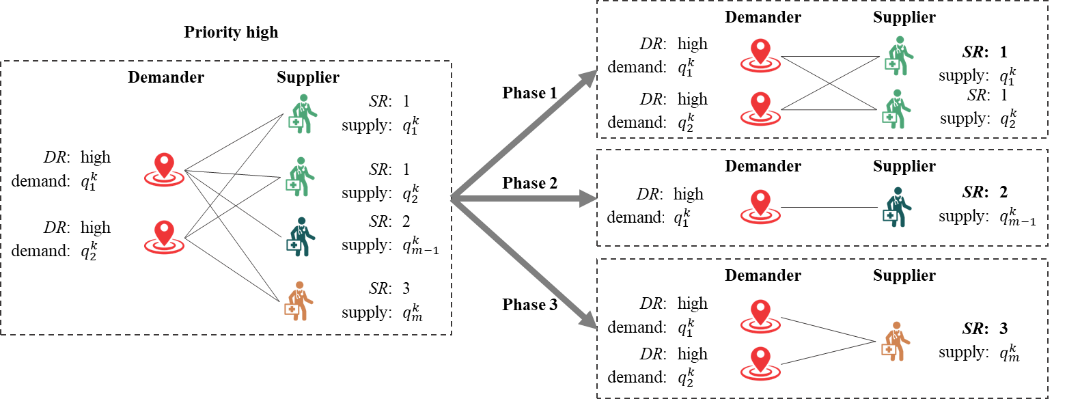


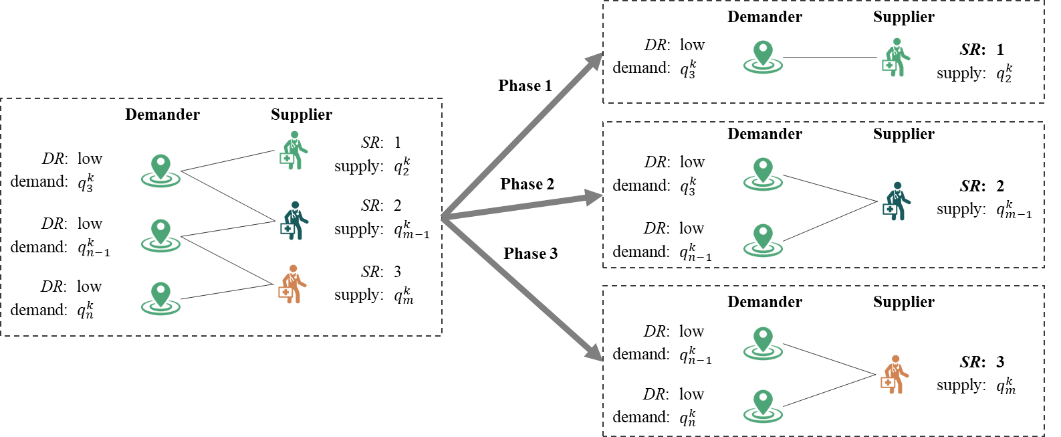


**Figure S4.** Divided the bipartite graph network according to the supply level of the supplier

**Supplementary Figure 5**

**Figure S5** illustrates the outcomes of the adaptive matching time window strategy. The x-axis is delineated into two segments: the first segment, ranging from 1 to 6, corresponds to the matching results under fully optimistic scenarios, while the second segment, spanning from 7 to 12, pertains to the matching results under partially optimistic scenarios. The data indicate that, in the majority of instances, the performance metrics—specifically average waiting time and matching rate—are superior in the partially optimistic scenarios relative to those observed in the fully optimistic scenarios.


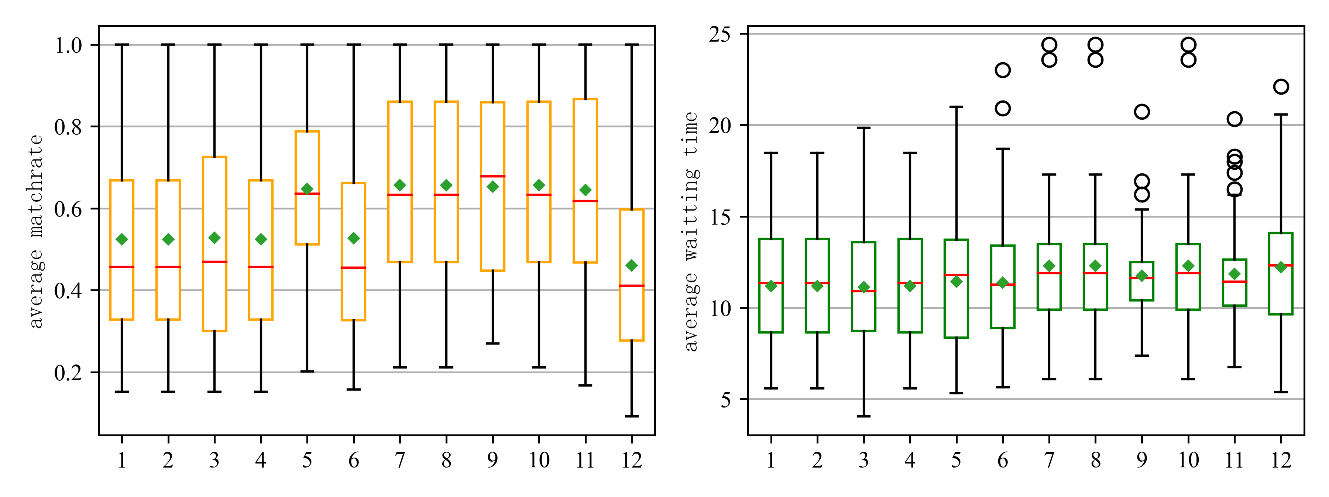


Figure S5. Results with adaptive batch sizes of the matching time window

# Supplementary Algorithms

**Supplementary Algorithm 1**

| **Algorithm S1:GMBMC algorithm** |
| --- |
| **Input：** $G_{PH}<U,V,E(U,V)>$   **Output：**  The maximum matching set ***M***, the average waiting time set ***AT***, and the average matching rate ***RT***.  **Algorithm**   1. for $i\in U$ do: 2. if $E(i,j)\neq-1,\text{ }j\in V$and sort by $E(i,j)$ from nearest to farthest 3. if $q_{i}==0$then 4. break 5. end if 6. for $j\in L_{i}$ do : 7. if $q_{j}==0$ do: 8. remove $j$ from $L_{i}$, continue 9. end if 10. if $q_{i}\leq q_{j}$then 11. ,,, 12. else 13. , , , , remove $j$ from $L_{i}$ 14. end if 15. , 16. end for 17. end for 18. return ***M*, *AT, RT*** |

Firstly, sort all suppliers $j$ that can match the recipient $i$ from nearest to farthest based on the Manhattan distance $l_{ij}$, and generate a set $L_{i}$. Then, prioritize the nearest suppliers from set $L_{i}$ to match with the recipient $i$ until the required quantity $q_{i}$ is satisfied, resulting in a matching set $M$. Furthermore, calculate the average matching rate $RT$ and the average waiting time $AT$. Finally, output $M$, $AT$, and $RT$.

**Supplementary Algorithm 2**

| **Algorithm S2:HMBMC algorithm** |
| --- |
| **Input：** $G_{PL}<U,V,E(U,V)>$   **Output：**  The maximum matching set ***M***, the average waiting time set ***AT***, and the average matching rate ***RT***.  **Algorithm**   1. for $i\in U$ do: 2. while $q_{i}>0$ do: 3. if $getPath(i,q_{i})$==false then 4. *break* 5. end if 7. , 8. end while 9. end for 10. return ***M*, *AT, RT*** |
| **function** :   1. , if $E(i,j)\neq-1,\text{ }j\in V$and sort by $E(i,j)$ from nearest to farthest 2. for $j\in L_{i}$ do 3. if $q_{j}$==0 then 4. remove $j$ from $L_{i}$, continue 5. end if 6. if $q_{i}\leq q_{j}$ then 7. , , 9. return true 10. else: 11. , , 13. if $j^{M}==j$ 14. for $u\in MV$do: 15. if $u==i$ or $E(u,j)\leq E(i,j)$ then 16. continue 17. end if 18. if $q_{i}<q_{_{u}}^{matched}$ then $getPath(u,q_{i})$ 20. else: $getPath(u,q_{u}^{matched})$ 22. end if 23. ,,update 24. , 26. return true 27. end for 28. end if 29. end for 30. return false   end **function** |

In the Hungarian-based many-to-many bipartite matching algorithm with capacity (see Algorithm S2), the study defines a recursive function $getPath(i,q_{i})$ to identify augmenting paths. For all matching demanders $i$ in ***U***, if the requested quantity $q_{i}>0$, then input $i$ and $q_{i}$ into the matching function $getPath()$. After executing the function, if the return value is true, update the matching quantity $q_{i}^{*}$, and calculate the average matching rate ***RT*** and the average waiting time ***AT*.** Continue looping until the return value of $getPath(i,q_{i})$ is false or $q_{i}=0$, at which point the loop will terminate. In function $getPath()$, sort all suppliers that can match the demanders from nearest to farthest according to $l_{ij}$, and generate a set $L_{i}$. Next, iterate over the elements in $L_{i}$. If the requested quantity $q_{i}$ is less than the supply quantity $q_{j}$, directly match $i$ and $j$; otherwise, match the remaining supply quantity of $j$ and recursively query the augmented path. At last, output $M$, $AT$, and $RT$.
